# Characteristics of survivors enrolled in the World Trade Center Health Program

**DOI:** 10.1080/19338244.2024.2410495

**Published:** 2024-10-31

**Authors:** Ruiling Liu, Albeliz Santiago-Colón, Emma Butturini, Travis L. Kubale, Joan Reibman

**Affiliations:** a World Trade Center Health Program, National Institute for Occupational Safety and Health (NIOSH), Centers for Disease Control and Prevention (CDC), Atlanta, GA, USA;; b New York University Langone Medical Center, New York, NY, USA

**Keywords:** World Trade Center Health Program, WTC-related health conditions, WTC survivors, WTC youth

## Abstract

The World Trade Center (WTC) Health Program is a limited federal health care program that provides medical monitoring and treatment for WTC-related health conditions to responders and survivors impacted by the terrorist attacks on September 11, 2001. This study described the characteristics of the Program survivor members (who lived, worked, went to school, daycare or adult daycare or present in the New York City Disaster Area of 9/11/2001) to stimulate innovative ideas for improving healthcare services, generate new research interest, and serve as a reference for future research on this population. Administrative and medical claims data collected from the Program start date (07/01/2011) through 2022 were used. As of 12/31/2022, there were 37,384 enrolled survivors: 5.0% were aged ≤21 years on 9/11/2001, 45.9% females, and 31.2% non-Hispanic Whites. A total of 24,148 (64.6%) were certified for at least one WTC-related condition, including neoplasms (36.0%), aerodigestive disorders (35.6%) and mental health conditions (18.6%); 22.9% were certified for more than one category. Certification rates of some WTC-related conditions differed by sex, age and race/ethnicity. WTC survivor population is diverse in sex, age and race/ethnicity, with a high proportion certified for certain WTC-related health conditions, providing great opportunities for research in various areas.

## Introduction

The terrorist attacks on September 11, 2001 (9/11) on the World Trade Center (WTC) exposed hundreds of thousands of people in New York City (NYC) to harmful environmental exposures and traumatizing events. It was estimated that more than 400,000 individuals were exposed to the WTC collapse and its aftermath.^1^

Early studies funded by Centers for Disease and Control (CDC) and performed by the New York State Department of Health and collaborating academic institutions, demonstrated new onset respiratory symptoms in residents.^2,3^ The CDC and the NYC Department of Health and Mental Hygiene established the WTC Health Registry to conduct public health surveys to understand the short- and long-term health effects caused by the 9/11 attacks among responders (workers and volunteers who performed rescue, recovery, clean-up, and other related support services in the disaster area) and survivors (community members).^1^ In 2006, NYC announced funding for the WTC Environmental Health Center (EHC) to provide treatment for survivors.^4^ The Consolidated Appropriations Act of 2008 subsequently provided federal funding for screening, diagnostic, and treatment services to survivors.^5^ The James Zadroga 9/11 Health and Compensation Act of 2010 (Zadroga Act)^1^ was signed into law in January 2011, creating the WTC Health Program (the Program), a limited federal health care program administered by NIOSH. The Program provides no-cost medical monitoring and treatment for responder members. For survivor members, the Program provides no-cost initial health evaluations (IHEs) and follow-up annual monitoring exams (AMEs) for eligible individuals, and it is the final payor after private or public insurance for treatment of WTC related health conditions. A timeline of key healthcare-related activities and programs supporting responders and survivors in the history of the WTC Health Program has been described in detail elsewhere.^5,6^

WTC Health Program Survivors are defined as those who were enrolled in the EHC Community Program before the Zadroga Act (pre-Zadroga members) and subsequent enrollees meeting eligibility requirements in the Zadroga Act (post-Zadroga members).^7^ Enrollment in the Program after the Zadroga Act requires exposure to the 9/11 attacks and its aftermath as a resident, building occupant, local worker, student, or having been in the New York City Disaster Area (NYCDA) and report symptoms of a WTC-related health condition.^7^ Exposure is defined by meeting criteria based on time and activity within the NYCDA as described in Table 1. The NYCDA is the area of Manhattan south of Houston Street, and any block in Brooklyn wholly or partially contained within a 1.5-mile radius of the former WTC complex in NYC. WTC-related health conditions include cancer, aerodigestive disorders, mental health conditions, acute traumatic injuries, and musculoskeletal disorders that are associated with exposures to the 9/11 attacks. Note that in this report, the term ‘Survivor’ is used to refer to an individual that has already enrolled as a survivor member in the Program, while ‘survivor’ refers to an individual that meets the definition of the general survivor population exposed to the 9/11 attacks in the NYCDA.

Upon enrollment in the Program, Survivors are classified as screening-eligible, and are assigned to one of the two Clinical Centers of Excellence (CCEs) in the New York metropolitan area (NYMA): the WTC EHC in the NYC Health + Hospitals System (H + H) or William Street Clinic (WSC) if they live in NYMA, or to the Nationwide Provider Network (NPN), a network of affiliated providers located across the United States, if they live out of NYMA.

A screening-eligible Survivor can obtain one IHE per lifetime at a CCE or the NPN at no cost, to determine whether they have any WTC-related health conditions. The IHE is a comprehensive exam that is designed to evaluate medical conditions as specified in the Zadroga Act, and that can result from exposures from the 9/11 attacks. Each IHE includes completion of a set of detailed questionnaires evaluating 9/11 exposure, detailed medical history and the following examinations: laboratory analyses of blood, urinalysis, screening spirometry to assess lung function, chest radiography and electrocardiogram (for those over 40 years old when clinically indicated).^7^ After the IHE, if appropriate, submission is made to the Program for approval for a certification of the member’s condition. If the condition is approved by the Program, the member is categorized as certified-eligible. Under rare circumstances, a Survivor may obtain certification prior to completion of the IHE if specific requirements, including medical record documentation and other certification requirements are met; all pre-Zadroga Survivors are also categorized as certified-eligible.^7^

All certified-eligible Survivors are authorized to receive medically necessary treatment related to their certified condition(s) and follow-up AMEs. The AME reassesses for health complaints and the intervening medical history occurring since the IHE or the prior monitoring exam; it also evaluates a member’s mental health and includes the same examination components as the IHE.^7^

In addition to providing health surveillance, medical monitoring, diagnosis and treatment of certified WTC-related health conditions, the WTC Health Program also conducts research examining physical and mental health conditions that have been, or might be related to the 9/11 terrorist attacks, as well as diagnostic and treatment uncertainty.

This article describes the WTC Health Program Survivor population, including demographics, prevalence of certified WTC-related health conditions, and membership and certification trends. The purpose is to stimulate innovative ideas for improving healthcare services, generate new research interest, and serve as a reference for future research on the nuanced demographics and needs of this population.

## Methods

### Data sources

All WTC Survivor enrolled before 2023 were included in this analysis. We used administrative and medical claims data collected from the Program start date (07/01/2011) through 09/30/2023. Administrative data includes some demographic information (age, sex, preferred language, and state of current residence) and WTC-related health condition certification data. Self-reported race/ethnicity is collected at the IHEs and updated in AMEs. Certification data includes members’ WTC-related health conditions. Medical claims data includes information collected *via* the Centers for Medicare & Medicaid Service CMS-1500 Claim Form for professional claims, or the UB-04 Claim Form for institutional claims. We used medical claims data to identify members’ IHEs and follow-up AMEs, diagnostic, and treatment services paid by the Program.

### Data analysis

We described Survivors’ demographic characteristics, program service utilization, and prevalence of certified WTC-related health conditions. Neoplasm certifications were grouped using the National Cancer Institute Surveillance, Epidemiology, and End Results (SEER) neoplasm causes of death recode.^8^ Trends of survivor membership and WTC-related health condition certifications by year from 2011 to 2022 were also described. Total survivor membership in each year was defined as the total number of Survivors by the end of the measurement year excluding those known to be deceased before the measurement year. We defined ‘youth’ as persons ≤21 years on 9/11/2001. All the analyses were conducted using SAS software 9.4, Copyright © [2002–2012] by SAS Institute Inc.

### Human subjects’ protection

This activity was reviewed by CDC and was conducted consistent with all applicable federal law, regulations, and CDC policy (See e.g., 45 C.F.R. part 46.102(l)(2), 21 C.F.R. part 56; 42 U.S.C. §241(d); 5 U.S.C. §552a; 44 U.S.C. §3501 et seq; the Health Insurance Portability and Accountability Act of 1996 (“HIPAA”) (Pub. L. 104–191; 42 U.S.C. § 1320d), as modified, and the corresponding implementing regulations, including the Privacy, Security, Breach Notification, and Enforcement Rules (45 C.F.R. pts. 160, 162, and 164)). The work was determined to meet the requirements of public health surveillance as defined in 45 CFR 46.102(l)(2); therefore, informed consent was not required for this surveillance activity, and the Declaration of Helsinki was not applicable.

## Results

### WTC Survivors’ characteristics

There was a total of 37,384 Survivors as of 12/31/2022, including 36,059 living members whose average age was 62.4 years on 12/31/2022. Of all the Survivors, 5.0% were youth and 55.7% aged 22–45 years on 9/11/2001; 45.9% were females; 34.2% were Non-Hispanic White persons, and 84.8% reported English as their preferred language. As of the end of 2022, 72.1% of Survivors were living in New York State, followed by New Jersey (13.4%) and Florida (4.7%), and 63.4% of Survivors were assigned to the two Survivor CCEs in NYMA. See Table 2 for details of all enrolled Survivors by age on 9/11/2001 and by their currently assigned CCE/NPN.

### WTC Survivors’ health service utilizations paid by the Program

Overall, through 2022, a total of 26,588 (71.1%) Survivors completed an IHE (for post-Zadroga members) or their first AME (for pre-Zadroga members); 31.0% received at least one AME; 29.5% received diagnostic services (i.e., claims paid under diagnostic benefit plan) and 23.9% received medical treatment services paid by the Program (Table 2).

When stratifying by age on 9/11/2001, the youth group had higher proportions of females and Asian persons, lower proportions of non-Hispanic White persons and lower utilization of services paid by the Program, including IHE, AME, and diagnostic or medical treatment services (Table 2).

When stratifying by currently assigned CCE/NPN, CCE Survivors had higher proportions of females, race/ethnicity minorities (Hispanic, Non-Hispanic Asian or Black persons), and members who preferred a non-English language; they also had lower utilization rate of IHE or diagnostic health service paid by the Program (Table 2).

### WTC condition certifications among Survivors

**Figure 1.**
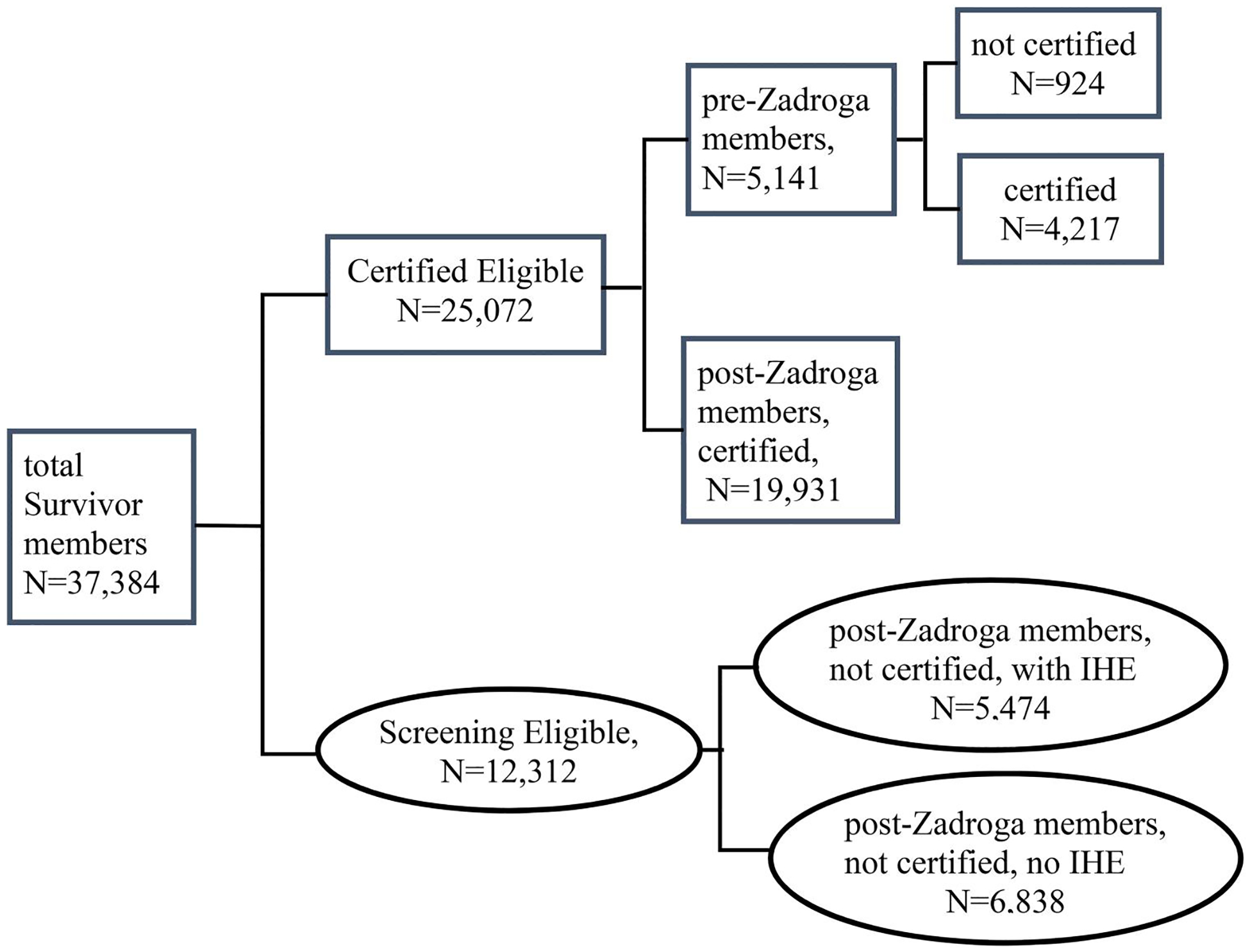
WTC Health Program Survivors by benefit category (screening eligible or certified eligible), as of 12/31/2022. Note: IHE, initial health evaluation. Screening-eligible Survivors include those who were enrolled after the Zadroga act (post-Zadroga members) and not certified for any WTC-related health conditions yet (with IHE or without IHE). Certified-eligible Survivors include Survivors enrolled before the Zadroga act (pre-Zadroga members) no matter certified or not and post-Zadroga members who had been certified for at least one WTC-related health condition

As of 12/31/2022, two thirds (*n* = 25,072) of Survivors were certified eligible, including all the 5,141 pre-Zadroga members (4,217 certified and 924 not certified) and 62% (*n* = 19,931) of post-Zadroga members who were certified for at least one WTC condition. The remaining one third (*n* = 12,312) were screening eligible; 56% (*n* = 6,838) of whom had no IHE yet. See Figure 1.

Overall, 64.6% of Survivors were certified for at least one WTC-health related condition through 2022 (Table 2). More than one third of Survivors were certified for neoplasms and aerodigestive disorders, respectively, and 18.6% were certified for at least one mental health condition. Close to one quarter (*n* = 8,553) of Survivors were certified in two or more categories; specifically, 12.5% (*n* = 4,688) were certified for at least one aerodigestive disorder and one mental health condition, 11.0% (*n* = 4,097) were certified for at least one aerodigestive disorder and a neoplasm, 5.0% (*n* = 1,850) were certified for at least one mental health condition and a neoplasm, and 2.5% (*n* = 920) were certified for a neoplasm, aerodigestive disorder and mental health conditions.

**Figure 2.**
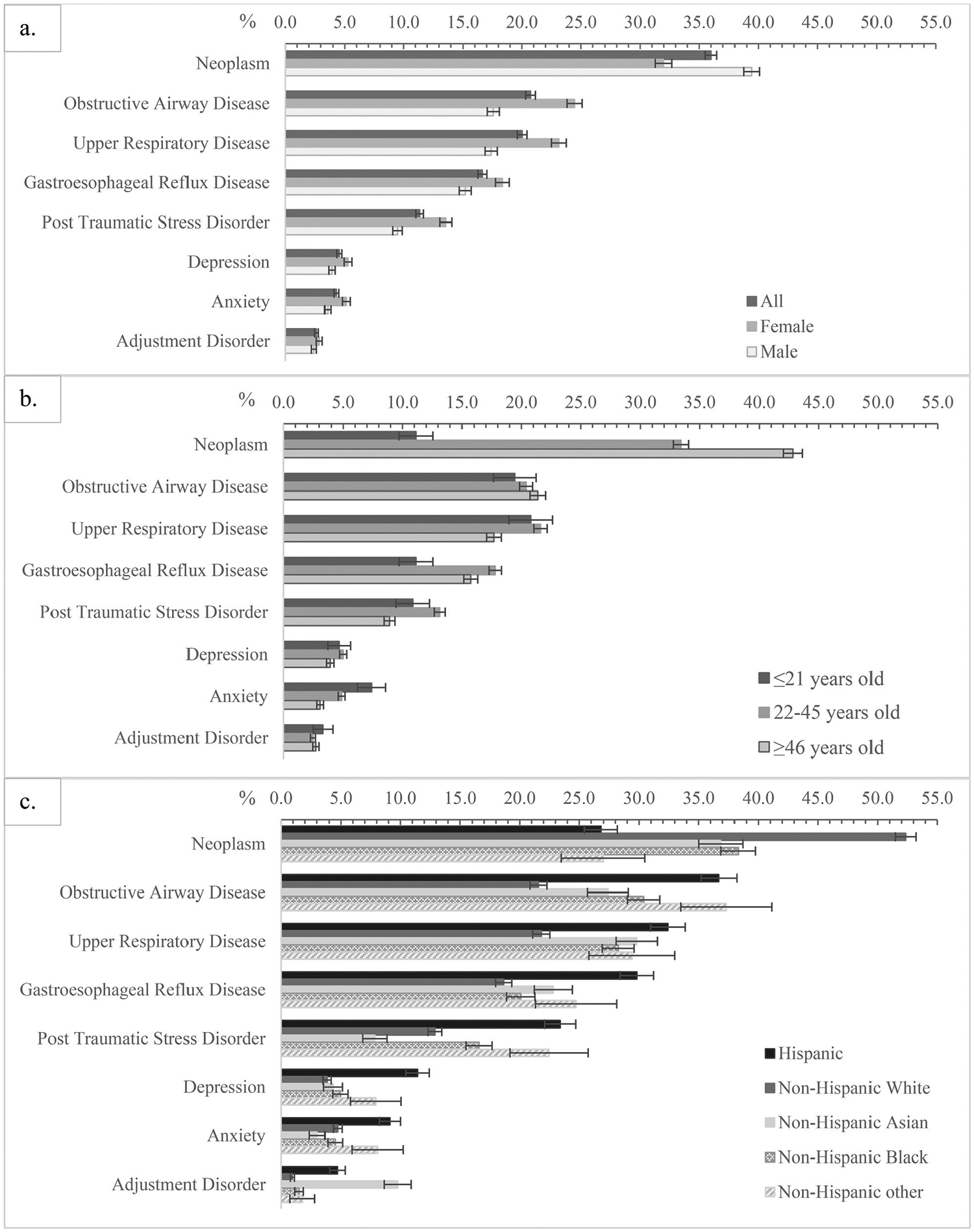
Certification rate of selected WTC-related health conditions among Survivors as of 12/31/2022, by members’ sex (a), age on 9/11/2001 (b), and race/ethnicity (c). Note: error bars show the 95% confidence intervals estimated with Wald tests. Obstructive airway disease includes chronic obstructive pulmonary disease (new onset or WTC-exacerbated), asthma, chronic cough syndrome, chronic respiratory disorder and reactive airways dysfunction syndrome. Upper respiratory disease includes chronic laryngitis, chronic nasopharyngitis, chronic rhinosinusitis, and upper airway hyperreactivity.

Certification rates differ by sex, age, and race/ethnicity (Figure 2). Rates were higher among women for obstructive airway disease (OAD, 24.5%), upper respiratory disease (URD, 23.1%), gastroesophageal reflux disease (GERD, 18.3%), post-traumatic stress disorder (PTSD, 13.6%), depression (5.3%) and anxiety (5.1%). However, women had lower certification rates for cancer (32.0%) than men (39.4%) (Figure 2(a)).

When stratified by age on 9/11/2001, youth had the lowest overall certification rate for any WTC conditions (48.2%, Table 2) but the highest certification rates for anxiety (7.4%). Those aged 22–45 years at exposure had the highest certification rates for GERD (17.8%) and PTSD (13.1%). Adults aged 46 years or older had the highest certification rates for cancer (42.8%) (Figure 2(b)).

When stratified by race/ethnicity, Hispanic persons had the highest certification rate for depression (11.4%) and GERD (29.8%). Non-Hispanic White persons had the highest certification rate for cancer (52.4%), Non-Hispanic Asian persons had the highest certification rate for adjustment disorder (9.8%). Hispanic persons and non-Hispanic minorities, compared to non-Hispanic White persons, had higher certification rates for OAD, URD and GERD (Figure 2(c)).

**Figure 3.**
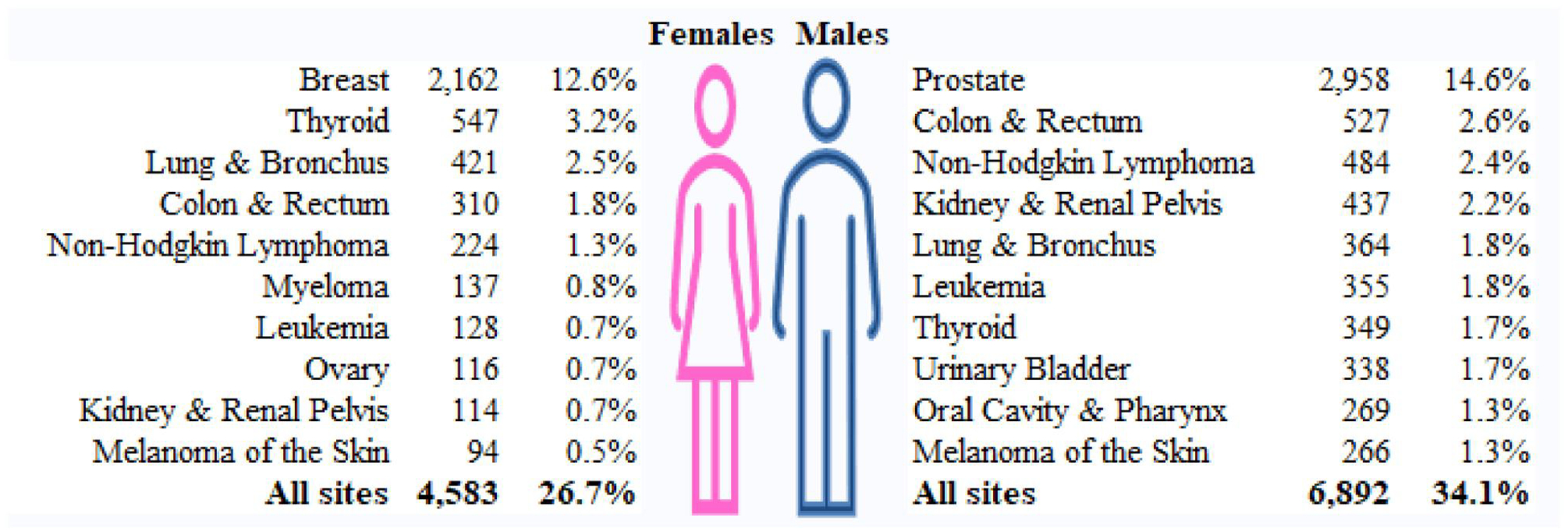
Top 10 certified cancer among Survivors as of 12/31/2022, by members’ sex. Note: The number and % show the number and percent of Survivors who have been certified for each cancer site; the denomination of the percentages are the total female Survivors (n = 17,162) for cancer among females and the total male Survivors (n = 20,222) for cancer among males. This figure only include cancer that are covered by the WTC Health Program as of 12/31/2022, excluding in situ, benign or unknown behavior neoplasms and non-melanoma of the skin cancer. Note that uterine cancer was added to the WTC condition list in 2023.

Figure 3 shows the top 10 certified cancers among Survivors by sex, excluding *in situ*, benign or unknown behavior neoplasms (women 820, men 809) and non-melanoma of the skin cancer (women 583, men 1,508). Note that uterine cancer was added to the WTC condition list in 2023 and thus was not included in this analysis. As of 12/31/2022, the most commonly certified cancer was breast cancer among women (12.6%) and prostate cancer among men (14.6%).

### Trends of Survivor membership and selected WTC condition certifications

**Figure 4.**
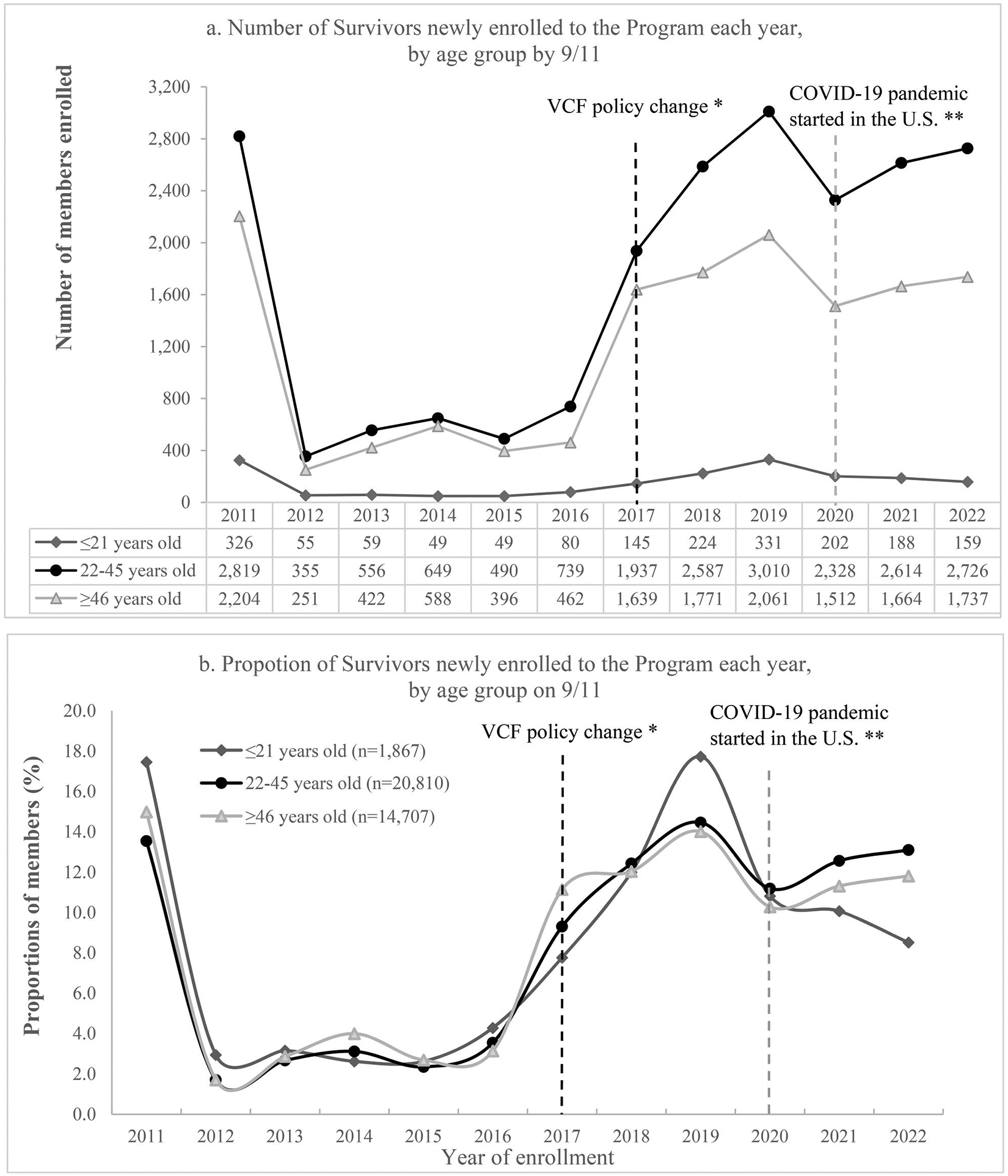
Enrollment trends of Survivors by age group on 9/11/2001, 2011–2022 Note: figure (a) shows the number of Survivors of each age group on 9/11/2001 that enrolled in each year; these are the numerators for figure (b). The denominators for figure (b) are the total number of Survivors in each group enrolled in all years from 2011 through 2022, i.e. for each Survivor group, percents of all years from 2011 through 2022 add up to 100%.*In 2017, Department of Justice’s September 11th Victim Compensation Fund (VCF) started to require claimants to be certified by the Program.^5^ **Coronavirus disease 2019 (COVID-19) pandemic started in the U.S.A in 2020. These two events have been noted in figures for context, although analyzing the impact of these specific events is beyond the scope of the paper.

Although the absolute numbers of Survivors enrolled to the Program varied markedly among age groups (Figure 4(a)), the overall temporal pattern of enrollment was similar (Figure 4(b)). A possible exception is a downward trend among youth between 2020–2022, where trends among the other age groups modestly increased. Overall, enrollment increased markedly from 2016 to 2019, then decreased in 2020 and started to increase again since 2021.

**Figure 5.**
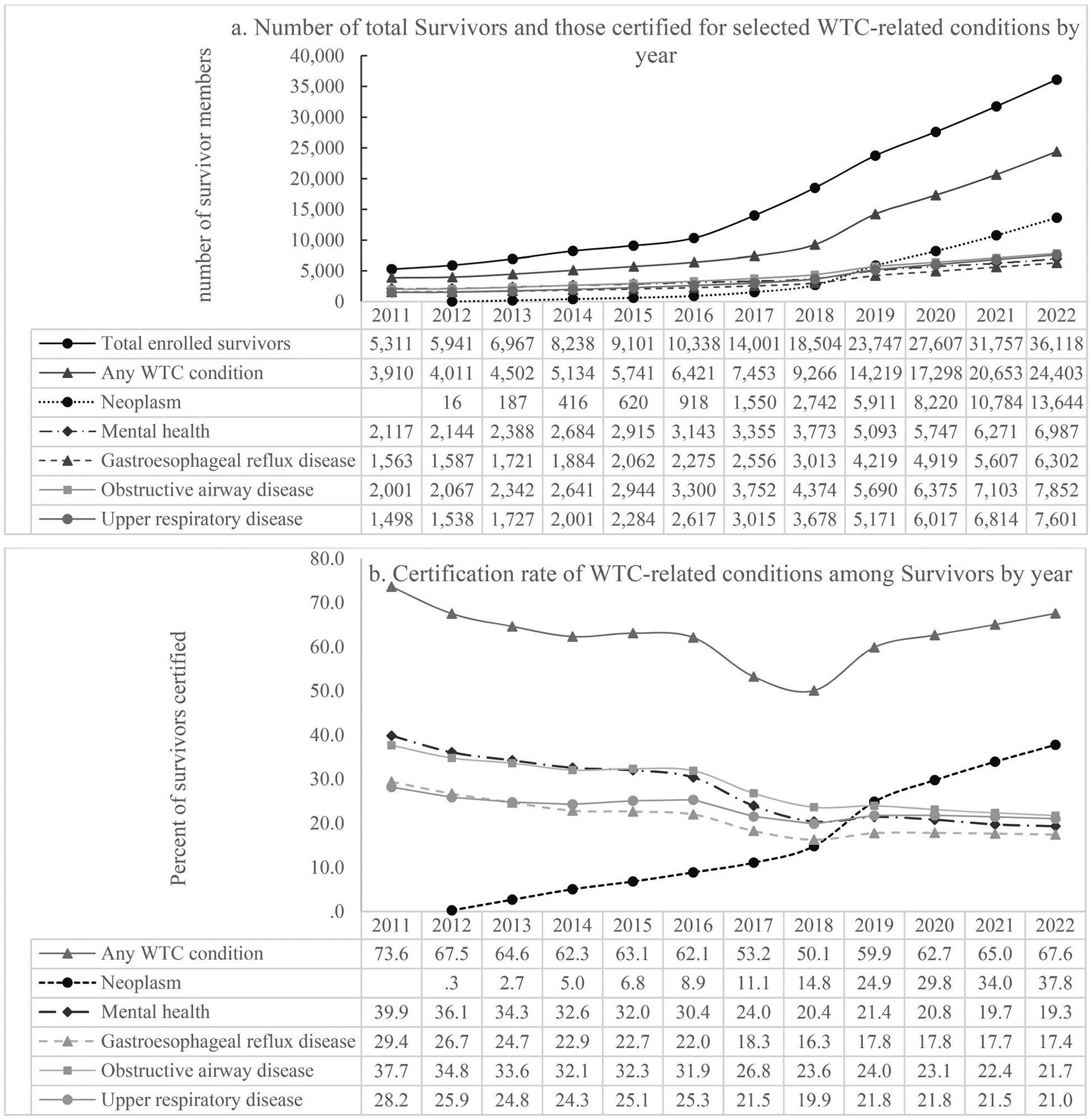
Survivor membership and proportions of Survivors certified for selected WTC-related conditions by year during 2011–2022. Note: 1. Figure a included total Survivors in the measurement year. Those who were known to have deceased in previous year(s) were not included in the measurement year. 2. Figure b, the denominators for the percentages were the total number of Survivors in the measurement year as shown in Figure a, and the numerators were the numbers of Survivors certified for the selected condition in the same year as shown in Figure a. 3. Neoplasms were added to the WTC condition list in Oct 2012. Mental health conditions include PTSD, depression, adjustment disorder, anxiety and substance abuse. Obstructive airway diseases include asthma, chronic respiratory disorder – fumes/vapors, and WTC-exacerbated COPD. Upper respiratory diseases include sleep apnea, chronic rhinosinusitis, chronic nasopharyngitis, chronic laryngitis, and upper airway hyperreactivity.

The total Survivor membership almost doubled from 2011 (*n* = 5,311) to 2016 (*n* = 10,338) and increased nearly six-fold by 2022 (*n* = 36,118) (Figure 5(a)). A similar trend was observed for the total number of Survivors certified for any WTC condition, which increased 60% from 2011 (*n* = 3,910) to 2016 (*n* = 6,421), and five-fold by 2022 (*n* = 24,403).

The total number of Survivors certified for neoplasm increased dramatically from 16 in 2012 (neoplasm was added to the WTC condition list in Oct 2012) to 187 in 2013 to 13,644 in 2022 (Figure 5(a)). The total number of Survivors certified for other types of WTC conditions also increased over the years, but less rapidly compared to neoplasm. See Figure 5(a) for details.

Certification rate of any WTC conditions among Survivors decreased from 73.6% in 2011 to 50.1% in 2018 and then increased to 66.9% in 2022; certification rate for neoplasm continued increasing from 2.7% in 2013 to 37.8% in 2022. However, certification rate for other WTC conditions, i.e., mental health conditions, GERD, OAD or URD, has been decreasing from 2011 to 2022 (Figure 5(b)) due to a more rapid increase in overall Survivor membership than in members certified for these conditions (Figure 5(a)).

## Discussion

A subset of the WTC Health Program survivor population has been described previously.^9^ This paper is the first to provide a comprehensive description of the Survivor population. The goal is to stimulate more research in various areas and to provide reference for future research of this population. However, it should be noted that the cohort description may change over time due to natural changes in enrollment demographics and potential changes in member eligibility (e.g., increased enrollment *via* the NPN, inclusion of new health conditions, and changes in policy).

### WTC Survivors’ characteristics

Survivor membership has increased dramatically through 2022, especially since 2017. This could be due in part to a policy change of the Department of Justice’s September 11th Victim Compensation Fund (VCF) in 2016. The VCF was created to provide financial compensation for any eligible individual (or a personal representative of a deceased individual) who suffered physical harm or was killed due to exposure to the 9/11 attacks.^10^ In 2016, the VCF started to require that claimants be enrolled in and certified for treatment by the WTC Health Program for specified physical conditions when filing for compensation. This change likely caused the sharp increase in Program’s enrollment after 2016. The decrease in enrollment observed in 2020 was likely attributed to the Coronavirus Disease 2019 (COVID-19) pandemic.

As of 2022, more than 30% of the Program members were Survivors, and the remaining were responder members. The number of enrollees increased more rapidly for survivors than responders.^11,12^ Compared with responder members, Survivors are more diverse in sex, age, and race/ethnicity distribution.^13^ Responder members were mostly White males at working age on 9/11, while Survivors have a much higher proportion of 9/11 females (45%), minority groups, youth, and older adults.

Survivors are also geographically diverse. Although as of 2022, 85% of Survivors lived in New York and New Jersey, more members are moving out of the two states in recent years, possibly due to a growing proportion reaching retirement age or rising living costs in the area. There are members living in almost every state of the United States.

The diversity of the WTC survivor population provides opportunities for research examining the impacts of the 9/11 attacks along with social determinants of health and lifestyle factors on health and quality of life on a wide spectrum of individuals, exploring health equity issues, and improving health care of potentially underserved populations. Research on the survivor population funded by the Program has led to hundreds of publications.^14^ The Program continues to solicit stakeholder inputs for research proposals that include potentially vulnerable populations, such as women, minorities, and individuals exposed to 9/11 as children.

### WTC Survivors’ service utilization

Initial health exams (IHEs) and annual monitoring exams (AMEs) are a key part of the WTC health surveillance activities. This analysis showed that only 71.1% of Survivors have completed an IHE or AME with the Program, and less than half of the certified-eligible Survivors had any AMEs as of 12/31/2022. These findings suggest the need for research regarding barriers to accomplishing IHEs/AMEs (e.g., system barriers, health behavior barriers) and methods to reduce these obstacles including outreach strategies to improve member engagement related to WTC health surveillance activities.

Certified-eligible Survivors can receive medical treatment for their WTC-related conditions; however, less than half of these members had claims paid by the Program. There are two main reasons for lacking claims data on Survivors. First, the Program must follow a coordination of benefits (COB) process for treatment and medication costs of a certified WTC-related health condition. COB requires Survivors to have primary health insurance and to submit claims to their primary insurance for payment first, with the Program being the last payor. This process likely results in incomplete utilization information maintained by the Program. Second, the absence of claims data could be due to members not having received medical care, which could occur for various reasons, such as the disease is under control and needs no treatment, members’ preference of not getting medical treatment or limited access to care. Member level factors such as stage of the health conditions, language barriers, socioeconomic status, geographic locations, and structural factors such as shortage of providers specializing in WTC conditions like mental health, or accepting WTC Health Program coverage, can all contribute to varied healthcare utilization. Future research can examine barriers related to access and timeliness of care.

### WTC-related health conditions among Survivors

Certification rates or prevalences of WTC-related conditions among Survivors are generally higher than the prevalences of corresponding conditions among the general U.S. population. For example, among the U.S. population aged 65 years old or over, 18.4% of women and 22.0% of men were alive with cancer on Jan 1, 2020,^15^ while 24.9% and 29.8% of the living Survivors had cancer in 2019 and 2020, respectively; the lifetime prevalence of PTSD among U.S. adults was 6.1%,^16^ while more than 10% of Survivors were certified for PTSD. However, the Survivor population is self-selected and eligibility for cohort entry is linked to having reported symptoms of a WTC-related condition. Therefore, comparisons with the general population are subject to selection bias. Given a highly selected population, identifying adequate external control groups is challenging but essential for unbiased comparisons. For this reason, existing research of survivors has favored cross-sectional, interventional, and other study designs including internal comparisons.

As expected, cancer prevalence among Survivors has been increasing as this population is aging, currently becoming the most frequently certified condition among Survivors. The top 10 most commonly certified cancers among men and women mirror the most common cancers diagnosed among men and women among the general population, respectively, in the U.S. in 2022.^17^ More detailed characteristics of survivors with cancer have also been described in previous publications with similar, although not identical distributions.^18–20^ However, these studies are generally descriptive and have not explicitly examined cancer risk among survivors, primarily because it is challenging to identify a comparable control group from the general population. Given the diverse makeup compared with other 9/11-exposed populations, the survivor population offers unique opportunities for future cancer research.

Elevated lower and upper airway symptoms were also noted among survivors soon after the attacks.^3,13,21^ Jordan and coauthors reported a higher age-adjusted lifetime prevalence of asthma (24.7%) for WTC Health Registry enrollees than the prevalence estimates reported on the 2015 National Health Interview Survey (12.7%) and the 2014 New York City Community Health Survey (11.3%).^22^ For some survivors, many conditions have resolved in the years following onset;^23^ however, longitudinal studies have reported persistent conditions for some aerodigestive disorders.^22^ Our analysis showed that more than one-third of the Survivors had at least one certified aerodigestive disorders, of which OAD, URD and GERD certification rates all exceeded 15%. Lung function tests are an integral component of the IHEs and AMEs and the longitudinal lung function data provided by these monitoring exams serves as a rich resource of future studies.

Mental health conditions associated with the 9/11 attacks are also common among survivors. Among the 18% Survivors who were certified for a mental health condition, PTSD is most frequently certified. Studies examining PTSD make up a large portion of the mental health research on survivors, with general agreement that there was a substantial PTSD burden and evidence of a dose-response relationship related to 9/11 exposure.^24,25^ Anxiety and adjustment disorders were commonly certified mental health conditions. Yet, the proportion of Survivors who have been certified for mental health conditions might be underestimated because some members may be reluctant to come forward.^26^ However, members’ mental health is monitored *via* questionnaire surveys at each IHE or AME. These monitoring data provide potential to further our understanding of co-morbid mental health conditions in the survivor population.^27^

Prevalence of some WTC conditions among Survivors differ by sex, age group or race/ethnicity, which generally mirror what has been observed among the general population or other populations. For example, women, compared to men, have a lower prevalence of cancer^15^ and higher prevalence of OADs like asthma^28,29^ and chronic obstructive pulmonary disease (COPD),^30^ URDs like chronic rhinosinusitis,^31^ GERD^29^ and PTSD;^32^ youth adults, compared to older adults, have lower risk for cancer overall and GERD, which are age related; and non-Hispanic White persons have the highest cancer incidence rate.^33^ Our analyses also showed that Hispanic and non-Hispanic minorities had higher prevalence for aerodigestive conditions than non-Hispanic White persons, the underlying driving factors are unknown and are worthwhile to explore in future studies on the survivor population.

### Research implications

The Program has been authorized through 2090 and survivor membership is increasing yearly. Given the unique exposure to the 9/11 attacks, the increasing prevalence of cancer and the potential for late onset of new health conditions among Survivors, a strong research program is clearly needed to elucidate health risks and improve treatment and diagnostic capabilities moving forward. The diversity of the survivor population is appealing for studying disease risk and trajectory, including issues of disparities in WTC-related health condition diagnosis, certification, and health care access among the survivor population. Future research findings may help identify new conditions that are associated with exposures to the 9/11 attacks, support program improvements, promote policies and strategies focused to provide equitable health care to all Survivors, enhance intervention treatment effectiveness, and strengthen existing best practices for diagnosing and treating WTC-related conditions.^34^

The Program is actively working to encourage new and innovative research on emerging areas of interest and engagement of new researchers. WTC Health Program research funding opportunity announcements have incorporated stakeholder recommendations on the research needs of the survivor population, including women, minorities, and individuals exposed to 9/11 as children. Information related to extramural research awarded by the WTC Health Program, to date, and the associated research publications are available as a resource to both concerned community members and potential research partners.^35^ Potential research funding applicants can also explore the 9/11 related research library that includes publications focusing on a variety of health outcomes among the survivor population to help them understand what has been studied to date and areas for further scientific exploration.^14^

## Limitations

There are notable limitations in our analysis. First, race/ethnicity information was missing for about one-third of Survivors; race/ethnicity information is collected or updated during their IHE and AME, and nearly 30% of enrolled survivors members have never had an IHE. As such, findings by race/ethnicity are less certain, and prevalence estimates for WTC conditions may be biased. Second, due to the nature of the claims process, there is a lag between claims submission and processing after a patient’s visit. The Program has a timely filing limit of 18 months from date of services for Survivors. As such, the extent of medical services, such as IHE, AME, and diagnosis/treatment services, may have been underestimated in 2022. Importantly, due to coordination of benefits, some Survivors may have their diagnosis/treatment services paid by their other health insurance, leading to underestimates of diagnosis/treatment services related to WTC conditions. Lastly, this analysis was not able to compare the incidence rate or prevalence of WTC conditions with the general population, as this population is self-referred, and it is out of the scope of this analysis to identify a sufficiently similar reference group to facilitate external comparisons of disease prevalence.

## Conclusion

WTC Survivors are diverse in age, sex, race/ethnicity, and geographic locations. Health conditions among these groups vary, suggesting the potential for inequities among underserved populations. Currently, few methods are available to systematically determine whether there are unique vulnerabilities and health disparities among survivors exposed to the 9/11 attacks and its aftermath. Research is needed to better understand differences in health conditions and healthcare engagement among potentially under-represented populations, such as ethnic/racial groups, women, and persons who were exposed to 9/11 as children. In addition, the Program’s surveillance activities have identified high prevalence of certain WTC-related chronic health conditions in the Survivors population like cancer, aerodigestive disorder and mental health conditions. Continuing research can elucidate patterns of late occurring and persistent physical and mental health conditions related to 9/11-exposure, further describe characteristics of certifiable conditions, and identify any new conditions that may arise.

## Figures and Tables

**Table 1.** Eligibility for individuals present in the New York City Disaster area (NYCDA)^1^ to enroll as Survivor members of the World Trade Center health Program.

Activity	Time period	Minimum time requirements
Present in the dust or dust cloud	9/11/2001	Not applicable
Worked, lived, attended school, childcare, or adult day care	9/11/2001 – 01/10/2002 9/11/2001 – 07/31/2002	≥4 h each day for ≥4 days ≥4h each day for ≥30 days
Worked as a cleanup worker or performed maintenance with extensive exposure to WTC dust, but activities were not related to support services in responding to the 9/11 event.	9/11/2001 – 01/10/2002	≥4h
Deemed eligible to receive a grant from the Lower Manhattan Development Corporation Residential Grant Program, who had a lease for a residence or bought a residence in the New York City disaster area, and who lived in that residence	9/11/2001 – 05/31/2003	Resided during the time frame
A person whose place of employment–was located in the NYCDA^1^; and was deemed eligible to receive a grant from the Lower Manhattan Development Corporation WTC Small Firms Attraction and Retention Act program or other government incentive program designed to revitalize the lower Manhattan economy after the September 11, 2001, terrorist attacks.	9/11/2001 – 05/31/2003	Located in the NYC disaster area during the time frame

Note: * See 42 CFR Part §88.8.

1 The area of Manhattan south of Houston Street, and any block in Brooklyn wholly or partially contained within a 1.5-mile radius of the former WTC complex in NYC.

Source: https://www.cdc.gov/wtc/eligiblegroups.html#nycSurvivor

**Table 2.** Characteristics of Survivors enrolled in the World Trade Center health Program as of Dec 31, 2022.

			Age on 9/11/2001						Current CCE/NPN^a^			
	All		< =21 years		22–45 years		≥46 years		CCE		NPN	
	Num	%	Num	%	Num	%	Num	%	Num	%	Num	%
All	37,384	100.0	1,867	100.0	20,810	100.0	14,707	100.0	23,713	100.0	13,671	100.0
Vital status												
Deceased	1,325	3.5	10	0.5	422	2.0	893	6.1	840	3.5	485	3.6
Living	36,059	96.5	1,857	99.5	20,388	98.0	13,814	93.9	22,873	96.5	13,186	96.5
Sex												
Female	17,162	45.9	988	52.9	9,982	48.0	6,192	42.1	12,121	51.1	5,041	36.9
Male	20,222	54.1	879	47.1	10,828	52.0	8,515	57.9	11,592	48.9	8,630	63.1
Race/ethnicity combined												
Hispanic	2,651	7.1	222	11.9	1,385	6.7	1,044	7.1	2,299	9.7	352	2.6
Non-Hispanic Asian	4,362	11.7	151	8.1	2,508	12.1	1,703	11.6	3,151	13.3	1,211	8.9
Non-Hispanic Black	4,022	10.8	243	13.0	2,563	12.3	1,216	8.3	3,041	12.8	981	7.2
Non-Hispanic White	12,774	34.2	454	24.3	6,670	32.1	5,650	38.4	6,699	28.3	6,075	44.4
Non-Hispanic other^b^	619	1.7	52	2.8	358	1.7	209	1.4	439	1.9	180	1.3
Unknown	12,956	34.7	745	39.9	7,326	35.2	4,885	33.2	8,084	34.1	4,872	35.6
Preferred language												
English	31,689	84.8	1,551	83.1	17,819	85.6	12,319	83.8	18,307	77.2	13,382	97.9
Other^c^	1,024	2.7	31	1.7	484	2.3	509	3.5	943	4.0	81	0.6
unknown	4,671	12.5	285	15.3	2,507	12.1	1,879	12.8	4,463	18.8	208	1.5
Most recent residential state												
New York	26,939	72.1	1,474	79.0	14,660	70.5	10,805	73.5	22,275	93.9	4,664	34.1
New Jersey	5,021	13.4	98	5.3	3,072	14.8	1,851	12.6	1,015	4.3	4,006	29.3
Florida	1,773	4.7	37	2.0	920	4.4	816	5.6	116	0.5	1,657	12.1
Pennsylvania	546	1.5	27	1.5	317	1.5	202	1.4	67	0.3	479	3.5
Connecticut	499	1.3	22	1.2	312	1.5	165	1.1	69	0.3	430	3.2
Other states	2,606	7.0	209	11.2	1,529	7.4	868	5.9	171	0.7	2,435	17.8
Enrollment time												
Pre-Zadroga act	5,141	13.8	308	16.5	2,730	13.1	2,103	14.3	4,890	20.6	251	1.8
Post-Zadroga act	32,243	86.3	1,559	83.5	18,080	86.9	12,604	85.7	18,823	79.4	13,420	98.2
Certified for WTC conditions^d^	24,148	64.6	900	48.2	13,360	64.2	9,888	67.2	16,193	68.3	7,955	58.2
Neoplasm	13,460	36.0	208	11.1	6,954	33.4	6,298	42.8	7,774	32.8	5,686	41.6
Aerodigestive disorders	13,322	35.6	586	31.4	7,606	36.6	5,130	34.9	10,241	43.2	3,081	22.5
Mental health conditions	6,952	18.6	394	21.1	4,347	20.9	2,211	15.0	5,087	21.5	1,865	13.6
Musculoskeletal disorder or acute traumatic injuries	44	0.1			30	0.1	14	0.1	27	0.1	17	0.1
Certified for ≥2 categories of WTC conditions	8,553	22.9	272	14.6	4,945	23.8	3,336	22.7	6,215	26.2	2,338	17.1
Neoplasm and mental health conditions	1,850	5.0	35	1.9	1,124	5.4	691	4.7	1,151	4.9	699	5.1
Neoplasm and aerodigestive disorder	4,097	11.0	47	2.5	2,074	10.0	1,976	13.4	2,935	12.4	1,162	8.5
Aerodigestive and mental health conditions	4,688	12.5	222	11.9	2,962	14.2	1,504	10.2	3,529	14.9	1,159	8.5
Neoplasm, aerodigestive and mental health conditions	1,047	2.8	16	0.9	611	2.9	420	2.9	705	3.0	342	2.5
With an IHE or AME^e^	26,588	71.1	1,102	59.0	15,006	72.1	10,480	71.3	15,840	66.8	10,748	78.6
With any AMEs	11,590	31.0	367	19.7	6,630	31.9	4,593	31.2	7,433	31.4	4,157	30.4
With medical claims related diagnostic	11,024	29.5	323	17.3	6,386	30.7	4,315	29.3	5,821	24.6	5,203	38.1
With medical claims related to treatment	8,939	23.9	305	16.3	5,248	25.2	3,386	23.0	6,128	25.8	2,811	20.6

Note:

a CCE, Clinical Center of Excellence; NPN: Nationwide Provider Network.

b Non-Hispanic other include American Indians or Alaskan Natives (*n* = 48), 29 Native Hawaiian/Pacific Islanders (*n* = 29), 153 multi-racial Survivors (*n* = 153) and other races without specifications (*n* = 389).

c Other languages include Chinese (*n* = 782), Spanish (*n* = 228) and Polish (*n* = 14).

d A member could be certified for more than one type of WTC conditions. Neoplasm: including 903 individuals within situ, benign or unknown behavior neoplasms. Aerodigestive disorders include obstructive airway disease, upper respiratory disease, gastroesophageal reflux disease, interstitial lung disease, and sarcoid; mental health conditions including post-traumatic stress disorder, depression, anxiety, adjustment disorder and substance abuse.

e IHE: initial health evaluation; AME: annual monitoring exam (*n* = 1,796).

## Footnotes

1. Title I of Pub. L. 111–347, as amended by Pub. L. 114–113, Pub. L. 116–59, and Pub. L. 117–328 codified at 42 U.S.C. § 300mm *et seq*.
